# Development and Validation of a Pre-Transplant Risk Score (LT-MVI Score) to Predict Microvascular Invasion in Hepatocellular Carcinoma Candidates for Liver Transplantation

**DOI:** 10.3390/cancers17091418

**Published:** 2025-04-24

**Authors:** Quirino Lai, Timothy M. Pawlik, Suela Ajdini, Jean Emond, Karim Halazun, Arvinder S. Soin, Prashant Bhangui, Tomoharu Yoshizumi, Takeo Toshima, Marlene Panzer, Benedikt Schaefer, Maria Hoppe-Lotichius, Jens Mittler, Takashi Ito, Etsuro Hatano, Massimo Rossi, Albert C. Y. Chan, Tiffany Wong, Chao-Long Chen, Chih-Che Lin, Alessandro Vitale, Laurent Coubeau, Umberto Cillo, Jan P. Lerut

**Affiliations:** 1 General Surgery and Organ Transplantation Unit, AOU Policlinico Umberto I, Sapienza University of Rome, 00185 Rome, Italy; ajdini.1797976@studenti.uniroma1.it (S.A.); massimo.rossi@uniroma1.it (M.R.); 2 Department of Surgery, The Ohio State University Wexner Medical Center, Columbus, OH 43210, USA; tim.pawlik@osumc.edu; 3 The New York Presbyterian Hospital, Columbia University, New York, NY 10032, USA; 4 Department of Surgery, Division Hepatobiliary and Pancreatic Surgery, NYU Langone Medical Center, New York, NY 10006, USA; 5 Medanta Institute of Liver Transplantation and Regenerative Medicine, Medanta-The Medicity, Gurgaon 122001, Indiapbhangui@gmail.com (P.B.); 6 Department of Surgery and Science, Kyushu University, Fukuoka 819-0395, Japan; yoshizumi.tomoharu.717@m.kyushu-u.ac.jp (T.Y.); toshima.takeo.962@m.kyushu-u.ac.jp (T.T.); 7 Department of Medicine I, Gastroenterology, Hepatology and Endocrinology, Medical University of Innsbruck, 6020 Innsbruck, Austriabenedikt.schaefer@i-med.ac.at (B.S.); 8 Klinik für Allgemein-, Viszeral- und Transplantationschirurgie, Universitätsmedizin Mainz, 55131 Mainz, Germany; maria.hoppe-lotichius@unimedizin-mainz.de (M.H.-L.); jens.mittler@unimedizin-mainz.de (J.M.); 9 Division of Hepato-Biliary-Pancreatic and Transplant Surgery, Department of Surgery, Graduate School of Medicine, Kyoto 606-8303, Japan; itotaka@kuhp.kyoto-u.ac.jp (T.I.); etsu@kuhp.kyoto-u.ac.jp (E.H.); 10 Department of Surgery, Queen Mary Hospital, The University of Hong Kong, Hong Kong, China; acchan@hku.hk (A.C.Y.C.); wongtcl@hku.hk (T.W.); 11 Department of Surgery, Kaohsiung Chang Gung Memorial Hospital, College of Medicine, Chang Gung University, Kaohsiung 83301, Taiwan, Chinaimmunologylin@gmail.com (C.-C.L.); 12 Department of Surgery, Oncology and Gastroenterology, University of Padua, 35122 Padua, Italy; alessandro.vitale@unipd.it (A.V.);; 13 Institut de Recherche Clinique, Université Catholique de Louvain, 1348 Brussels, Belgium; laurent.coubeau@saintluc.uclouvain.be (L.C.); jan.lerut@uclouvain.be (J.P.L.)

**Keywords:** living-donor liver transplantation, alpha-fetoprotein, recurrence, tumor burden score

## Abstract

**Background/Objectives:** MVI is a relevant prognostic factor among patients with hepatocellular carcinoma (HCC) receiving liver transplantation (LT). The preoperative assessment of the risk for MVI is relevant to pre-LT patient management and selection. The objective of this study was to create and validate a model to predict microvascular invasion (MVI) based on preoperative variables in the LT setting. **Methods**: A total of 2170 patients from 11 collaborative centers in Europe, Asia, and the US, who received transplants between 1 January 2000 and 31 December 2017, were enrolled in the study. The entire cohort was split into a training and a validation set (70/30% of the initial cohort, respectively) using random selection. **Results**: MVI was reported in 586 (27.0%) explanted specimens. Using the training set data, multivariable logistic regression identified three preoperative parameters associated with MVI: α-fetoprotein (lnAFP; odds ratio [OR] = 1.19; 95% confidence interval [CI] = 1.13–1.27), imaging tumor burden score (lnTBS; OR = 1.66; 95%CI = 1.39–1.99), and a fast-track approach before LT due to the availability of a live donation (OR = 1.99; 95%CI = 1.56–2.53). In the validation set, the LT-MVI c-index was 0.74, versus 0.69 for the MVI score proposed by Endo et al. (Brier Skill Score +75%). The new score had a relevant net reclassification index (overall value = 0.61). Stratifying the validation set into three risk categories (0–50th, 51st–75th, and >75th score percentiles), a very good stratification was observed in terms of disease-free (5-year: 89.3, 75.5, and 50.7%, respectively) and overall survival (5-year: 79.5, 72.6, and 53.7%, respectively). **Conclusions**: The preoperative assessment of MVI using the proposed score demonstrated very good accuracy in predicting MVI after LT.

## Simple Summary

Microvascular invasion (MVI) is an important factor that affects the prognosis of patients with hepatocellular carcinoma (HCC) undergoing liver transplantation. Identifying patients at high risk for MVI before surgery can help doctors to make better treatment decisions and improve outcomes. This study aimed to develop and validate a predictive model for MVI using preoperative clinical and imaging data. By analyzing data from 2170 liver transplant patients across multiple international centers, the researchers identified three key factors linked to MVI: α-fetoprotein levels, tumor burden on imaging, and the urgency of transplant due to a living donor. The new predictive score showed strong accuracy in classifying patients into different risk groups, helping to estimate long-term survival after transplantation. This tool may assist transplant teams in selecting candidates and tailoring treatment strategies to improve patient outcomes.

## 1. Introduction

Liver transplantation (LT) is the most effective curative treatment for carefully selected patients with hepatocellular carcinoma (HCC) [1]. However, 10–15% of patients experience recurrence following LT [2]. Numerous studies have explored the impact of various clinicopathological factors on postoperative recurrence [3,4,5]. Among these, microvascular invasion (MVI) has been identified as a key predictor of poor post-transplant survival [6,7].

Unfortunately, MVI can only be definitively diagnosed through pathological examination of the resected specimen, limiting its preoperative assessment [8]. As a result, there is significant interest in developing accurate preoperative tools to predict MVI [9,10,11]. Such tools could assist clinicians in tailoring treatment strategies for LT candidates, optimizing organ allocation, refining donor–recipient matching, and adjusting surgical approaches [12,13].

Several preoperative models for predicting MVI have been proposed, incorporating biological markers (e.g., serum alpha-fetoprotein [AFP], neutrophil-to-lymphocyte ratio [NLR]) and morphological characteristics (e.g., tumor size and number, tumor burden score [TBS]) [14,15,16]. However, most models have been derived from data on patients undergoing hepatic resection rather than LT. A recent study by Endo et al. introduced an accessible online calculator for predicting MVI [16]. However, unlike resection cohorts, LT candidates typically undergo extensive evaluation, bridging treatments, and often experience selection pressure over time. These elements influence tumor biology and recurrence risk differently than in patients treated with resection. Furthermore, models like the one proposed by Endo et al. have not been calibrated on post-transplant pathology and outcomes, limiting their applicability in this setting.

As a consequence, the present study aimed to develop and validate a novel scoring system—the LT-MVI score—to predict the risk of MVI in LT candidates. This new score was compared with previously published MVI prediction models designed for hepatic resection.

## 2. Materials and Methods

The local ethics board of Sapienza University of Rome, Italy, approved the current study. Informed consent for personal data was obtained from all patients at the time of the transplant; however, no consent was obtained for this study, because of its retrospective nature. The Strengthening the Reporting of Observational Studies in Epidemiology (STROBE) reporting guidelines were followed.

### 2.1. Study Design, Setting, and Population

In this multicenter, retrospective cohort study, the data of adult patients (aged ≥ 18 years) undergoing LT for the primary diagnosis of HCC were investigated. All participating centers had relevant experience in LT and transplant oncology and are part of the EurHeCaLT and West–East Collaborative Liver Transplant study groups. Specifically, the 11 centers that made up the cohort were located in Europe (Université Catholique de Louvain, Brussels, Belgium; Sapienza University of Rome, Rome, Italy; University of Padua, Padua, Italy; University of Innsbruck, Innsbruck, Austria; Universitätsmedizin Mainz, Mainz, Germany), Asia (Medanta–The Medicity, Delhi, India; University of Hong Kong, Hong Kong, China; Kyoto University, Kyoto, Japan; Chang Gung Memorial Hospital, Kaohsiung City, Taiwan; Kyushu University, Fukuoka, Japan), and North America (Columbia University, New York, NY, USA). The cohort comprised 2170 HCC patients who received transplants between 1 January 2000 and 31 December 2017. The exclusion criteria were retransplantation, post-LT diagnosis of mixed hepatocellular–cholangiocellular cancer, or cholangiocellular cancer misdiagnosed as HCC.

### 2.2. Outcomes, Data Collection, and Definitions

The primary outcome was MVI, which was identified in the pathological specimen after LT. The last follow-up date was 31 December 2019. Data were retrospectively retrieved from prospectively maintained databases comprising transplant centers from the EurHeCaLT and West–East Collaborative Liver Transplant study groups. Q.L., the data manager of the study groups, served as the guarantor of the data quality. When possible, data errors and missingness across the databases were identified and solved with specific queries to the centers.

The total tumor burden score (TBS) was adopted as a metric used to evaluate the overall burden of HCC. TBS was calculated based on the size and number of tumors present, and it provided a quantitative measure of tumor burden. The TBS was calculated using the following formula:

*TBS* = √ (*D*2 + *N*2)

where *D* is the maximum diameter of the largest tumor (in cm) at last radiology, and *N* is the number of tumors at last radiology.

The upper limit of the tumor burden for LT differed among the participating centers. From 2000 onward, the initially applied restrictive Milan criteria were progressively abandoned. A more conservative approach was reported in the case of deceased-donor LT, for which the University of California San Francisco and the Up-to-Seven restriction criteria were primarily used. No morphological limitations were frequently adopted in living-donor LT (LDLT), except for the presence of major vascular invasion and extrahepatic disease localization.

The “fast-track approach” for live donation refers to cases where patients underwent liver transplantation via a living donor within a short wait time (<2 months), often bypassing bridging therapies due to donor availability.

### 2.3. Statistical Analysis

Continuous variables were reported as medians and first–third quartiles (Q1–Q3). Categorical variables were reported as numbers and percentages. Continuous variables were compared using the Mann–Whitney U test; categorical variables were compared using the chi-square test or Fisher’s exact test, as appropriate. Survival probabilities were compared using the log-rank test and estimated using the Kaplan–Meier method. In all cases, missing data relative to the study covariates involved less than 10% of patients and were handled with a single imputation method. Specifically, a median of nearby-points imputation was adopted. The median instead was adopted of the mean due to the potentially skewed distribution of the managed variables.

The entire cohort was initially divided to generate a training and a validation set (70/30% of the initial cohort, respectively), using purely random selection. Univariable analysis was performed to analyze the training set data to explore the impact of the investigated preoperative variables on the risk of MVI. A natural logarithm transformation was performed on variables with a markedly skewed distribution. Variables with a *p*-value ≤ 0.20 on univariable analysis were initially included in the multivariable analysis. A multivariable logistic regression analysis with backward Wald exclusion was used to create a parsimonious model in terms of the number of covariates. Odds ratios (ORs) with 95% confidence intervals (95%CIs) were presented. The b-coefficients of the selected significant values in the final step of the multivariable analysis were used to develop a weighted risk score.

The discriminative performance of the derived score was subsequently evaluated for the validation set using the area under the receiver operating characteristic (ROC) curve (AUC). The discriminatory ability of the new LT-MVI score was tested against three previously published scores [14,15,16].

The Eastern Hepatobiliary Surgery Hospital (EHBH)-MVI score was calculated according to the formula AFP (≤20 ng/mL = 0; 20–400 ng/mL = 1; >400 ng/mL = 2) + tumor encapsulation (complete = 0, incomplete = 1, absent = 2) + tumor diameter (≤5 cm = 0, >5 = 2) + HBeAg (negative = 0, positive = 1) + HBV DNA load (≤104 IU/mL = 0, >104 IU/mL = 2) + number of tumors (solitary = 0, multiple = 2) + gastric fundus/esophagus varicosity (absent = 0, present = 1) [14].

The nomogram proposed by Lei et al. was calculated using the beta coefficients of the following variables: large tumor diameter, multiple tumors, incomplete tumor capsule, presence of typical dynamic pattern, AFP, platelet count, and HBV DNA load [15].

The MVI score developed by Endo et al. was calculated using the equation −1.798 + 0.442 × (lnAFP) + 0.102 × (TBS at last imaging) + 0.158 × NLR [16].

Different cut-off values were explored to estimate low and high risk for MVI. The diagnostic odds ratio (DOR) was used to explore the cut-offs corresponding to different deciles and quartiles. The Brier Score and the Brier Skill Score were reported to examine the additive accuracy to predict the risk of MVI provided by the new score. A negative value of the Brier Skill Score corresponded to inferior performance of the tested model with respect to the presently proposed score, considered as the reference score. The model’s reliability was tested on the validation set, comparing expected versus observed survival. The Hosmer–Lemeshow test assessed the calibration between the observed and expected event rates. Bar calibration plots were constructed to visually represent the agreement between the predictions and observations in each risk group. The net reclassification (NRI) was defined as the proportion of patients with increased or decreased predicted risk of MVI, comparing the new MVI score versus the one proposed by Endo et al. [16]. Patients with an increased risk according to the new model were considered to be upgraded, whereas patients with a reduced risk were considered to be downgraded. To quantify the model’s clinical utility, the net benefit was also calculated, to explore whether the benefits conferred by identifying the true positives outweighed the harms conferred by identifying the false positives. The net benefit was calculated using the following formula: ((True positives − False positives)/N) × (pt/(1 − pt)).

A *p*-value of <0.05 was considered significant for all analyses; all tests were 2-tailed. The SPSS statistical package version 27.0 (SPSS Inc., Chicago, IL, USA), R (version 4.1.2), and the STATA package version 16.0 (StataCorp LLC, College Station, TX, USA) were used for statistical analyses.

## 3. Results

Demographic and tumor-related characteristics of the entire population (*N* = 2170) and the training (*n* = 1519; 70.0%) and validation (*n* = 651; 30.0%) sets are summarized in Table 1.

The median (Q1–Q3) follow-up was 3.5 (1.5–6.4) years in the entire cohort. The median (Q1–Q3) age at LT was 59 (53–63) years, with most patients being male (*n* = 1719, 79.2%). The median waiting time was 3 (1–7) months, with 967 (44.6%) patients receiving an LDLT. The primary underlying liver disease was HCV-related cirrhosis (*n* = 1.015, 46.8%), followed by HBV-related cirrhosis (*n* = 582, 26.8%) and alcohol-related cirrhosis (*n* = 126, 19.4%). The median model for end-stage liver disease (MELD) value was 12 (8–16).

At the time of LT, 608 (28.0%) patients were radiologically outside of the Milan criteria, with a median TBS of 3.2 (2.0–4.6). The last available AFP value was 10 (440) ng/mL.

During the pre-LT period, 1629 (75.1%) patients received any neoadjuvant loco-regional therapy (median number of procedures for each patient = 2, Q1–Q3 = 1–3). In 259 (11.9%) cases, a complete response according to the modified Response Evaluation Criteria in Solid Tumors (mRECIST) was reported, followed by 514 (23.7%) cases with a partial response, 480 (22.1%) with stable disease, and 376 (17.3%) with progressive disease.

On pathology, more than one-quarter of patients had MVI (*n* = 586, 27.0%), 263 (12.1%) tumor specimens had a poor tumor grade, and 774 (35.7%) cases exceeded the conventional Milan criteria. In Supplementary Table S1, demographic and tumor-related characteristics of the patients with or without MVI are reported.

After splitting the entire cohort into validation and testing cohorts, no substantial differences were noted, except for a shorter waiting time observed in the validation set (*p* = 0.01). In addition, progressive disease according to mRECIST was also slightly more predominant among cases in the training set (18.4 versus 14.9%, *p* = 0.06).

### 3.1. LT-MVI Score Creation

Using data from the training set, a multivariable logistic regression model was constructed to assess the risk of MVI (Table 2).

Three different risk factors for MVI were identified: last available lnAFP before LT (OR = 1.19, 95%CI = 1.13–1.27, *p* < 0.001), lnTBS at last imaging before LT (OR = 1.66, 95%CI = 1.39–1.99; *p* < 0.001), and a fast-track approach before LT due to the availability of a live donation (OR = 1.99, 95%CI = 1.56–2.53; *p* < 0.001). According to the results observed, the following equation was created:

−2.500 + 0.507 *×* (lnTBS) + 0.178 *×* (lnAFP) + 0.686 *×* (LDLT)

**Figure 1:**
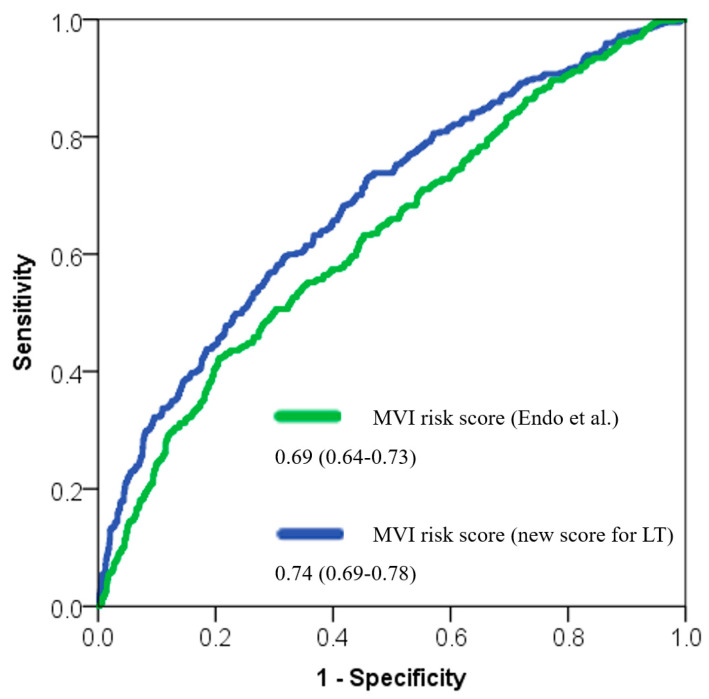
ROC curves comparing the discrimination ability of the LT-MVI score with respect to the score previously proposed by Endo et al. [16].

The new score performed well in the validation set to predict LT-MVI, with a very good AUC of 0.74 (95%CI = 0.69–0.78; *p* < 0.001), which was superior compared with the score developed by Endo et al. (AUC = 0.69, 95%CI = 0.64–0.73; *p* < 0.001) (Table 3, Figure 1).

The Brier Score and the Brier Skill Score were calculated to characterize the magnitude of prediction improvement using the new LT-MVI score. Based on the Brier Skill Score results, when considering the LT-MVI score (cut-off = 95th percentile) as the referral value, the newly proposed MVI risk tool demonstrated improved prediction relative to other scores, with a significant increase in the prediction accuracy compared with the score proposed by Endo et al. (+75%) (Table 3).

Three different cut-offs were examined in the LT-MVI score, with the intent of identifying three different risk classes: median value (predicted MVI risk = 23.6%), third quartile (predicted MVI risk = 32.7%), and 95th percentile (predicted MVI risk = 52.6%). The latter cut-off of the 95th percentile represented the best DOR (value = 5.85) among the values examined (Table 3).

### 3.2. Calibration of the LT-MVI Score

**Figure 2:**
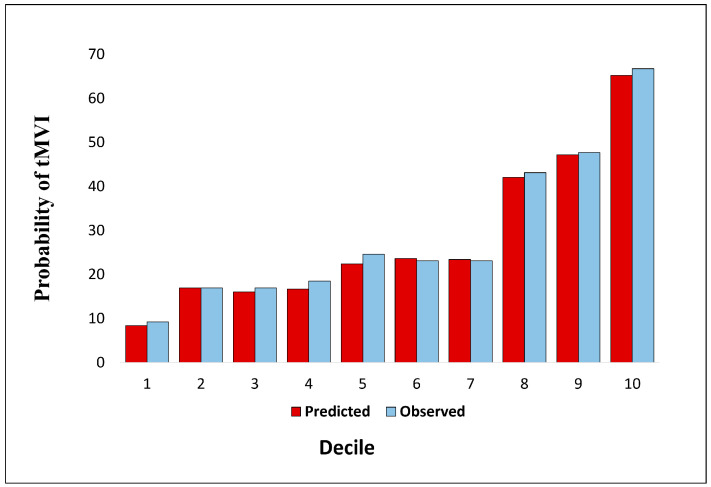
Bar plot showing the comparison between the predicted versus observed cases of MVI in the validation set population, stratified in deciles.

The Hosmer–Lemeshow test reported for the validation set showed that the prediction model had good calibration in the predicted versus observed MVI events (value = 0.67), as also depicted in a bar calibration plot (Figure 2). After stratifying the validation dataset’s population into deciles, the visual representation of the excellent agreement between predicted versus observed MVI cases was evident.

### 3.3. Net Reclassification Index and Net Benefit of the LT-MVI Score

Investigating the NRI in the validation set, the new LT-MVI score demonstrated a strong ability to reclassify patients relative to the risk of MVI, compared to the score proposed by Endo. Specifically, 122/189 patients with MVI were correctly reclassified with an increased predicted risk, while only 57/189 cases had a downstaged score. As for the non-MVI cases, 284/462 were correctly downstaged, and only 161/462 were incorrectly upstaged. The overall NRI was 0.610, which was consistent with an overall excellent ability to reclassify the risk class of the investigated patients correctly (Table 4). In examining the net benefit of the LT-MVI score, namely, the capacity to avoid futile transplantations due to a high risk of MVI, 1 in 24 transplantations could potentially be avoided using the 95th percentile cut-off. A lower cut-off demonstrated no net benefit or a minor impact (Table 4).

### 3.4. Survival

**Figure 3:**
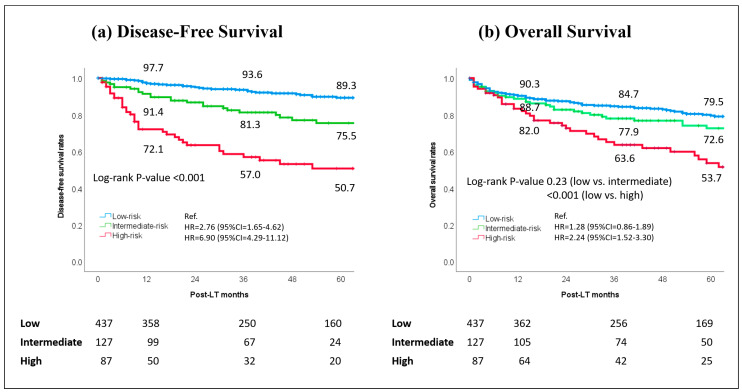
Disease-free survival and overall survival rates in the validation set: the population was stratified into three classes of risk, according to the LT-MVI.

As noted in Figure 3a, the model was able to stratify patients well in terms of DFS using the three risk classes. Specifically, patients at low risk had 1-, 3-, and 5-year DFS of 97.7%, 93.6%, and 89.3%, respectively, versus 91.4%, 81.3%, and 75.5% in intermediate- (log-rank *p* < 0.001) and 72.1%, 57.0%, and 50.7% (log-rank *p* < 0.001) in high-risk patients, respectively. Similar results were observed regarding overall survival (Figure 3b). Patients at low risk of MVI had markedly superior results compared with patients at high risk of MVI (log-rank *p* < 0.001). Specifically, the 1-, 3-, and 5-year overall survival was 90.3%, 84.7%, and 79.5% versus 82.0%, 63.6%, and 53.7%, respectively.

In addition, we explored survival outcomes among high-risk patients who did not undergo fast-track LDLT, stratified by a composite definition of tumor progression, integrating both radiological response and AFP kinetics. Patients with progressive disease and/or AFP progression >15 ng/mL/month had a significantly worse prognosis, with 1-, 3-, and 5-year DFS of 55.6% at all timepoints, compared to 88.9% in patients without signs of progression. The corresponding overall survival rates were 66.7%, 55.6%, and 55.6% versus 77.8% at all timepoints, respectively. These findings reinforce the prognostic value of dynamic biology assessment and support its integration into pre-transplant evaluation strategies for patients at high risk of MVI.

## 4. Discussion

In the current study, we have proposed a novel score based on preoperative factors to predict the risk of MVI in patients undergoing LT. This score effectively stratified patients at high risk of MVI and, consequently, those with a higher likelihood of worse long-term outcomes, such as poorer DFS and overall survival. Based on these predefined cut-offs, patients can be stratified into low, intermediate, and high MVI risk categories to guide treatment strategy and listing decisions.

The score integrates three key variables: AFP, TBS, and a fast-track approach before transplant due to the opportunity of an available live donation. From a practical standpoint, the LT-MVI score is readily usable in the clinical setting, because these three variables are easily obtainable before transplantation, without requiring sophisticated analyses or advanced methods of radiological interpretation.

AFP is a well-established risk factor for adverse outcomes in HCC patients. A large US study of 6817 HCC patients listed for LT demonstrated that AFP values at listing and the last pre-transplant measurement strongly predicted drop-out risk and intent-to-treat survival [17]. Similarly, in a multicentric European–Asiatic experience, AFP combined with tumor morphology formed the Metroticket 2.0 score, which was strongly associated with HCC-specific death (c-statistic = 0.78, 95%CI = 0.76–0.80; *p* < 0.001) [18]. Another study from the EurHeCaLT database, analyzing 306 Milan criteria IN and 116 Milan criteria OUT patients, found that an AFP slope >15 ng/mL/month and mRECIST tumor progression were independent predictors of HCC recurrence and post-LT mortality, regardless of the Milan criteria status [19].

TBS has also been linked to adverse outcomes in HCC. Representing a composite measure of the major lesion’s diameter and the number of nodules, TBS was initially developed for colorectal liver metastases [20] but was soon applied to HCC. A large ITA.LI.CA cohort (*N* = 4759) demonstrated that TBS was an independent predictor of overall survival, with each TBS point increase raising the risk of death by 6%. Patients with TBS ≥ 8, MELD ≥ 15, and AFP ≥ 1000 ng/mL had the worst long-term prognosis (*p* < 0.001) [21]. Additionally, an international study (*N* = 4089) assessing the HALTHCC score found that combining AFP, MELDNa, and TBS provided excellent MVI prediction (score 0–5 vs. >35: 7.7% vs. 70.6%; *p* < 0.001), with strong prognostic utility for recurrence (c-index = 0.71) and post-LT survival (C-index = 0.63) [22].

The association between live donation and MVI risk may seem paradoxical. Studies have shown that LDLT is correlated with better intent-to-treat [23] and post-LT survival [24] compared to deceased donation. However, LDLT recipients often present with more advanced tumors due to more permissive selection criteria [25]. A comparative study between Italian and Hong Kong data linked the increased post-LT HCC recurrence risk in LDLT to “salvage transplantation”, where patients with recurrent HCC post-resection undergo transplantation [26]. Negative features in the primary tumor often persist in the recurrent tumor [27], raising concerns about the optimal strategy for such cases [28]. Notably, both Western and Eastern studies have reported higher MVI rates in LDLT [29,30,31,32]. A US series of 36 LDLT cases found an MVI incidence of 58% [29], while another US study noted a difference of 22% vs. 10% between live- and deceased-donor LT [30]; similarly, a Hong Kong study reported 35% vs. 18% [31]. A key factor is that live donation shortens waiting times, reducing the “selection-by-time” effect. A US study of 6160 HCC patients listed for LT found that patients from regions with short waiting times (median: 1.6 vs. 7.6 months) received fewer loco-regional therapies and had worse survival (HR = 1.55, 95%CI = 1.38–1.74; *p* < 0.0001) [32]. A Canadian study (*N* = 851) confirmed that patients with a potential live donor received fewer neoadjuvant treatments (67.7% vs. 57.1%, *p* = 0.01) [33]. Thus, including “fast-track LT due to live donation availability” in the LT-MVI score is logical, as it likely reflects less aggressive pre-transplant management of biologically aggressive tumors, rather than an inherent risk from the LDLT procedure itself.

The LT-MVI score should impact clinical decision-making in several ways. Preoperative identification of candidates at high risk of microvascular invasion (MVI) allows for tailored management, including the adoption of intensified neoadjuvant strategies such as loco-regional therapies or emerging systemic options like immunotherapy.

As for the role of more aggressive neoadjuvant strategies in preoperatively identifying high-risk patients, a prospective US study showed that two tumor markers (PIVKA and AFP-L3) identified patients with an unacceptable post-LT recurrence risk, who may benefit from intensified loco-regional or systemic therapy pre-transplant [34]. The EurHeCaLT group demonstrated that up to three loco-regional treatments improved ITT survival in LT candidates meeting the Milan criteria [35]. Similarly, a US Multicenter HCC Transplant Consortium study confirmed this benefit in downstaged Milan criteria OUT patients, highlighting loco-regional therapies’ role in patient selection based on tumor biology [36].

The introduction of first-line systemic therapy with atezolizumab–bevacizumab has revolutionized advanced HCC treatment [37]. While post-LT immunotherapy is discouraged due to severe rejection and graft loss risks, recent reports suggest its potential in a neoadjuvant setting with proper patient selection and drug washout [38]. Therefore, preoperatively high-risk MVI patients may be ideal candidates for intensified loco-regional and systemic therapy, allowing for selection based on tumor aggressiveness [39].

Additionally, the score may influence listing policies, donor selection, and allocation strategies by stratifying patients not only by morphological criteria but also by biological aggressiveness.

To enhance clinical usability and promote widespread adoption, we are developing an online calculator for the LT-MVI score. This tool will allow for rapid, user-friendly computation based on input values for AFP, TBS, and LDLT status.

Some limitations should be noted. This multicentric retrospective study may be subject to biases related to time, geography, and center-specific differences in patient selection and tumor management. However, the inclusion of real-world data from high-volume transplant centers enhances its generalizability. With the intent to minimize information bias, we used prospectively maintained databases, implemented central data verification, and addressed missing covariate data using single median-based imputation.

Another limitation is the temporal constraint of the follow-up data, which ended in December 2019. While this timeframe allowed for sufficient follow-up to assess post-transplant outcomes, it may not reflect more recent trends in HCC management or transplant policies, including the evolving use of systemic therapies and changes in selection criteria.

Another limitation is that the score was not assessed in an ITT setting, as MVI evaluation was unavailable for patients who dropped out or died during the waiting period. Furthermore, other potential prognostic markers, such as AFP-L3 [34], PIVKA [40,41], donor type (brain vs. cardiac death), and preservation techniques (e.g., machine perfusion) [42], were not included, due to data unavailability. Future research should explore the role of genetic markers in refining prediction scores like the LT-MVI score to improve predictive performance.

## 5. Conclusions

A preoperative model incorporating AFP, tumor burden, and LDLT was developed to predict the risk of MVI in post-LT specimens. The LT-MVI score demonstrated strong accuracy, discrimination, calibration, and net reclassification ability. Implementing this predictive model may aid surgeons in patient selection and optimizing therapeutic strategies for HCC patients undergoing LT.

## Figures and Tables

**Table 1:** Demographic and tumor-related characteristics of the training set and validation set.

Variables	Entire Population (*N* = 2170; 100.0%)	Training Set (*n* = 1519; 70.0%)	Validation Set (*n* = 651; 30.0%)	*p*-Value
	Median (Q1–Q3) or *n* (%)			
Age, years	59 (53–63)	59 (53–64)	58 (54–63)	0.43
Male sex	1719 (79.2)	1199 (78.9)	520 (79.9)	0.64
Live donation	967 (44.6)	667 (43.9)	300 (46.1)	0.37
Waiting time duration, months	3 (1–7)	3 (1–8)	3 (1–7)	0.01
Underlying liver disease *				
HCV	1015 (46.8)	693 (45.6)	322 (49.5)	0.10
HBV	582 (26.8)	418 (27.5)	164 (25.2)	0.27
Alcohol	126 (19.4)	307 (20.2)	126 (19.4)	0.68
NASH	160 (7.4)	117 (7.7)	43 (6.6)	0.42
Other	114 (5.3)	80 (5.3)	34 (5.2)	1.00
Lab-MELD	12 (8–16)	12 (8–16)	12 (9–16)	0.30
Radiological features at entry				
Diameter of target lesion, cm	2.5 (1.8–3.5)	2.5 (1.8–3.8)	2.5 (1.0–3.0)	0.66
Number of nodules	1 (1–3)	1 (1–3)	1 (1–3)	0.22
Milan-OUT status	679 (31.3)	473 (31.1)	206 (31.6)	0.84
Radiological features at LT				
Diameter of target lesion, cm	2.2 (1.3–3.3)	2.2 (1.3–3.3)	2.1 (1.2–3.2)	0.60
Number of nodules	1 (1–3)	1 (1–3)	1 (1–3)	0.77
Milan-OUT status	608 (28.0)	424 (27.9)	184 (28.3)	0.88
TBS	3.2 (2.0–4.6)	3.2 (2.0–4.6)	3.2 (2.0–4.6)	0.99
LRT number of treatments	2 (1–3)	2 (1–3)	1 (0–3)	0.68
Radiological response mRECIST				
CR	259 (11.9)	185 (12.2)	74 (11.4)	0.61
PR	514 (23.7)	357 (23.5)	157 (24.1)	0.78
SD	480 (22.1)	321 (21.1)	159 (24.4)	0.09
PD	376 (17.3)	279 (18.4)	97 (14.9)	0.06
No LRT	541 (24.9)	377 (24.8)	164 (25.2)	0.87
AFP, ng/mL				
At entry	14 (5–55)	14 (5–55)	14 (6–56)	0.93
At LT	10 (4–40)	10 (4–38)	4 (10–44)	0.35
NLR at LT	2.8 (1.9–4.4)	2.8 (1.9–4.3)	2.9 (2.0–4.5)	0.52
PLR at LT	68 (39–107)	68 (38–108)	68 (41–104)	0.99
Pathological features				
Diameter of target lesion, cm	2.5 (1.5–3.5)	2.5 (1.5–3.5)	2.5 (1.5–3.5)	0.62
Number of nodules	2 (1–3)	2 (1–3)	2 (1–3)	0.38
Milan-OUT status	774 (35.7)	533 (35.1)	241 (37.0)	0.41
Poor grading	263 (12.1)	180 (11.8)	83 (12.7)	0.57
MVI	586 (27.0)	397 (26.1)	189 (29.0)	0.17

**Abbreviations:** MVI, microvascular invasion; *n*, number; Q1, first quartile; Q3, third quartile; %, percentage; HCV, hepatitis C virus; HBV, hepatitis B virus; NASH, non-alcoholic steatohepatitis; MELD, model for end-stage liver disease; LT, liver transplantation; TBS, tumor burden score; LRT, loco-regional therapy; mRECIST, modified Response Evaluation Criteria in Solid Tumors; CR, complete response; PR, partial response; SD, stable disease; PD, progressive disease; AFP, alpha-fetoprotein; NLR, neutrophil-to-lymphocyte ratio; PLR, platelet-to-lymphocyte ratio; MVI, microvascular invasion. * In some cases, the same patient presented multiple underlying liver diaseases.

**Table 2:** Univariable and multivariable analyses in the training set.

**Variable**	**Univariable Analysis**					**Multivariable Analysis**				
	**Beta**	**SE**	**OR**	**95%CI**	***p*** **-Value**	**Beta**	**SE**	**OR**	**95%CI**	***p*** **-Value**
Fast-track before LDLT	0.90	0.12	2.45	1.94–3.10	<0.001	0.69	0.12	1.99	1.56–2.53	<0.001
lnTBS at last imaging	0.68	0.09	1.97	1.65–2.34	<0.001	0.51	0.09	1.66	1.39–1.99	<0.001
lnAFP	0.23	0.03	1.26	1.19–1.34	<0.001	0.18	0.03	1.19	1.13–1.27	<0.001
NASH	0.54	0.20	1.72	1.16–2.55	0.007	-	-	-	-	-
Age, years	−0.02	0.007	0.98	0.97–1.00	0.007	-	-	-	-	-
Waiting time, months	−0.01	0.007	0.99	0.97–1.00	0.03	-	-	-	-	-
Lab-MELD	−0.02	0.01	0.98	0.96–1.00	0.06	-	-	-	-	-
lnPLR	0.11	0.06	1.11	0.99–1.25	0.07	-	-	-	-	-
Male sex	0.25	0.15	1.28	0.96–1.72	0.10	-	-	-	-	-
HBV	0.20	0.13	1.22	0.95–1.57	0.13	-	-	-	-	-
HCV	−0.15	0.12	0.86	0.68–1.08	0.19	-	-	-	-	-
Alcohol	−0.05	0.15	0.95	0.72–1.27	0.75	-	-	-	-	-
lnNLR	0.02	0.08	1.02	0.87–1.19	0.81	-	-	-	-	-
Constant	-	-	-	-	-	−2.50	0.16	0.08	-	<0.001

-Log2 likelihood: 1607.91; Hosmer–Lemeshow test *p*-value = 0.98. **Abbreviations:** SE, standard error; OR, odds ratio; CI, confidence intervals; LDLT, living-donor liver transplantation; TBS, tumor burden score; AFP, alpha-fetoprotein; NASH, non-alcoholic steatohepatitis; MELD, model for end-stage liver disease; PLR, platelet-to-lymphocyte ratio; HBV, hepatitis B virus; HCV, hepatitis C virus; NLR, neutrophil-to-lymphocyte ratio.

**Table 3:** Prognostic ability for the risk of MVI explored in the validation set.

Score		SE	AUC	95%CI	*p*-Value	Brier Skill Score	Brier Skill Score (%)
LT-MVI risk score		0.02	0.74	0.69–0.78	<0.001	0.2456	Ref.
MVI risk score by Endo et al. [16]		0.02	0.69	0.64–0.73	<0.001	0.4286	−0.75
Nomogram by Lei et al. [15]		0.02	0.69	0.65–0.73	<0.001	0.2618	−0.07
EHBH MVI score [14]		0.02	0.68	0.64–0.73	<0.001	0.2995	−0.22
**Different cut-offs of the LT-MVI score**							
**Decile**	**%**	**Sens**		**Spec**		**DOR**	
50	23.6	72.0		58.4		3.61	
75	32.7	58.2		77.3		4.74	
95	52.6	29.6		93.3		5.85	

**Abbreviations:** SE, standard error; AUC, area under the curve; CI, confidence interval; LT, liver transplantation; MVI, microvascular invasion; EHBH, Eastern Hepatobiliary Surgery Hospital.

**Table 4:** NRI and net benefit of the proposed score in the validation set.

NRI of the LT-MVI Score Versus the MVI Score Proposed by Endo et al. [16]
Events: (number of events with increased predicted risk − number of events with decreased predicted risk)/number of events 122-57/189 = 34.4%
Non-events: (number of non-events with decreased predicted risk − number of non-events with increased predicted risk)/number of non-events 284-161/462 = 26.6%
Overall NRI: event NRI + non-event NRI 0.344 + 0.266 = 0.610
**Net benefit**
50th percentile: (136–191)/651 × 0.236/(1 − 0.236) = −0.02610 (no net benefit) 75th percentile: (110–104)/651 × 0.327/(1 − 0.327) = 0.00448 (1 in 223) 95th percentile: (56–31)/651 × 0.526/(1 − 0.526) = 0.04262 (1 in 24)

**Abbreviations:** NRI, net reclassification index; LT, liver transplantation; MVI, microvascular invasion.

## Data Availability

The datasets generated and/or analyzed during the current study are not publicly available but are available from the corresponding author upon reasonable request.

## Supplementary Materials

The following supporting information can be downloaded at https://www.mdpi.com/article/10.3390/cancers17091418/s1, Table S1: Demographic and tumor-related characteristics of the investigated cohort.

## Abbreviations

The following abbreviations are used in this manuscript:

95%CI	95% Confidence interval
AFP	Alpha-fetoprotein
AUC	Area under the curve
DOR	Diagnostic odds ratio
HCC	Hepatocellular carcinoma
LDLT	Living-donor liver transplantation
EHBH	Eastern Hepatobiliary Surgery Hospital
HALTHCC	Hazard Associated with Liver Transplantation for Hepatocellular Carcinoma
LT	Liver transplantation
MELD	Model for end-stage liver disease
mRECIST	Modified Response Evaluation Criteria in Solid Tumors
MVI	Microvascular invasion
NLR	Neutrophil-to-lymphocyte ratio
NRI	Net reclassification
OR	Odds ratio
Q1–Q3	First–third quartiles
ROC	Receiver operating characteristic
STROBE	Strengthening the Reporting of Observational Studies in Epidemiology
TBS	Tumor burden score
