# Supplementary material for: Development and Validation of a Pre-Transplant Risk Score (LT-MVI Score) to Predict Microvascular Invasion in Hepatocellular Carcinoma Candidates for Liver Transplantation
Source: Cancers (Basel). 2025 Apr 24;17(9):1418. doi: 10.3390/cancers17091418 (PMC12070955; doi:10.3390/cancers17091418)
Supplement: Supplementary file 1 [file cancers-17-01418-s001.zip › cancers-3560558-supplementary.pdf]

SUPPLEMENTARY MATERIAL

Supplementary Table S1. Demographic and tumor-related characteristics of the investigated cohort.

Variables	No MVI (n=1,584; 73.0%)	MVI (n=586; 27.0%)	P-value
	Median (Q1-Q3) or n (%)		
Age, years	59 (54-64)	57 (53-63)	0.002
Male sex	1,235 (78.0)	484 (82.6)	0.02
Waiting time duration, months	3 (1-8)	2 (1-6)	<0.001
Potential live donation	610 (38.5)	357 (60.9)	<0.001
Underlying liver disease*			
HCV	745 (47.0)	270 (46.1)	0.70
HBV	413 (26.1)	169 (28.8)	0.21
Alcohol	318 (20.1)	115 (19.6)	0.86
NASH	102 (6.4)	58 (9.9)	0.007
Other	95 (6.0)	19 (3.2)	0.009
Lab-MELD	12 (9-16)	11 (8-15)	0.005
Radiological features at entry			
Diameter of target lesion, cm	2.5 (1.8-3.5)	2.9 (2.0-4.3)	<0.001
Number of nodules	1 (1-3)	2 (1-3)	<0.001
Milan-OUT status	426 (26.9)	253 (43.2)	<0.001
Radiological features at LT			
Diameter of target lesion, cm	2.0 (1.2-3.0)	2.7 (1.8-4.0)	<0.001
Number of nodules	1 (1-2)	2 (1-4)	<0.001
Milan-OUT status	357 (22.5)	251 (42.8)	<0.001
TBS	2.9 (1.7-4.2)	3.9 (2.6-5.8)	<0.001
LRT number of treatments	2 (1-3)	1 (0-3)	0.12
Radiological response mRECIST			
CR	226 (14.3)	33 (5.6)	<0.001
PR	406 (25.6)	108 (18.4)	<0.001
SD	340 (21.5)	140 (23.9)	0.24
PD	243 (15.3)	133 (22.7)	<0.001
No LRT	369 (23.3)	172 (29.4)	0.004
AFP, ng/mL			
At entry	12 (5-41)	23 (7-155)	<0.001
At LT	8 (4-26)	21 (6-151)	<0.001
NLR at LT	2.8 (1.9-4.4)	2.9 (1.9-4.6)	0.47
Pathological features			
Diameter of target lesion, cm	2.2 (1.4-3.2)	3.0 (2.0-4.5)	<0.001
Number of nodules	1 (1-3)	3 (1-5)	<0.001
Milan-OUT status	445 (28.1)	329 (56.1)	<0.001
Poor grading	135 (8.5)	128 (21.8)	<0.001

Abbreviations: MVI, microvascular invasion; n, number; Q1, first quartile; Q3, third quartile; %, percentage; HCV, hepatitis C virus; HBV, hepatitis B virus; NASH, non-alcoholic steatohepatitis; MELD, model for end-stage liver disease; LT, liver transplantation; TBS, total burden score; LRT, loco-regional therapy; mRECIST, modified Response Evaluation Criteria in Solid Tumors; CR, complete response; PR, partial response; SD, stable disease; PD, progressive disease; AFP, alpha-fetoprotein; NLR, neutrophil-to-lymphocyte ratio.
